# Factors Associated with Human Papillomavirus Vaccination Among Diverse Adolescents in a Region with Low Human Papillomavirus Vaccination Rates

**DOI:** 10.1089/heq.2018.0028

**Published:** 2018-09-01

**Authors:** Deanna Kepka, Julia Bodson, Djin Lai, Ana Sanchez-Birkhead, Jeannette Villalta, Valentine Mukundente, Fahina Tavake-Pasi, France A. Davis, Doriena Lee, Edwin Napia, Ryan Mooney, Heather Coulter, Louisa A. Stark

**Affiliations:** ^1^ Cancer Control and Population Sciences, Huntsman Cancer Institute, Salt Lake City, Utah.; ^2^ College of Nursing, University of Utah, Salt Lake City, Utah.; ^3^ Community Faces of Utah, Salt Lake City, Utah.; ^4^ Hispanic Healthcare Task Force, Salt Lake City, Utah.; ^5^ Best of Africa, West Valley City, Utah.; ^6^ National Tongan American Society, Salt Lake City, Utah.; ^7^ Calvary Baptist Church, Salt Lake City, Utah.; ^8^ Urban Indian Center, Salt Lake City, Utah.; ^9^ School of Medicine, University of Utah, Salt Lake City, Utah.; ^10^ Department of Human Genetics, School of Medicine, University of Utah, Salt Lake City, Utah.

**Keywords:** HPV vaccination, African American, African immigrant, Native American, Latino, Pacific Islander

## Abstract

**Introduction:** This study assesses the sociodemographic facilitators and barriers to human papillomavirus (HPV) vaccination for diverse teens in a region with low HPV vaccination rates.

**Materials and Methods:** In this community-based participatory research study, we surveyed adult family members of teens aged 11–17 years from African American, African refugee, American Indian/Alaskan Native, Hispanic/Latino, and Native Hawaiian/Pacific Islander community groups in Salt Lake City, Utah. Bivariate analyses assessed associations between sociodemographic characteristics and, respectively, HPV vaccine receipt and intentions for vaccination. Barriers to vaccination were also investigated.

**Results:** Only 20% of participants had vaccinated at least one of their children with at least one dose of the HPV vaccine. HPV vaccination was significantly related to caregiver age (*p*=0.035), race/ethnicity (*p*=0.001), educational attainment (*p*=0.006), annual household income (*p*=0.0454), years in the United States (*p*=0.023), and caregiver parent birthplace (*p*=0.008). Among caregivers with unvaccinated children, intention to vaccinate was significantly related to race/ethnicity (*p*=0.048 for daughters; *p*=0.003 for sons), caregiver parent birthplace (*p*=0.023 for sons), health insurance coverage (*p*=0.028 for daughters; *p*=0.047 for sons), and type of health insurance coverage (*p*=0.008 for sons). The most frequently cited barriers to HPV vaccination were lack of knowledge about the HPV vaccine, costs, side effects, and child not being sexually active.

**Conclusions:** Our results show substantially lower HPV vaccine coverage than both national and state rates, signaling the urgent need for multipronged HPV vaccination interventions within these communities; strategies are discussed.

## Introduction

Human papillomavirus (HPV) causes ∼31,500 cancers annually in the United States, including cervical, vulvar, anal, penile, and head and neck cancers.^1^ The HPV vaccine can prevent up to 73% of HPV-related diagnoses and 49% of HPV-related deaths^2^; yet nationally, 50.5% of female adolescents and 62.5% of male adolescents are not up to date on the vaccination series.^3^ Coverage in Utah is even worse, with most recent estimates showing that only 41.3% of female adolescents and 20.3% of male adolescents have completed the vaccination series.^3^

Although the Utah population has historically been predominately non-Hispanic white, the state is rapidly gaining racial/ethnic diversity, with about 20% of current residents identifying as Hispanic and/or non-white.^4^ Hispanics/Latinos are the largest minority group in the state (about 371,000 residents), comprising up to 33% of the population in certain cities.^5^ This population has grown rapidly in recent years, with the most recent census-to-census change (2000–2010) showing a 78% increase.^5^ Another large minority group in Utah is the American Indian/Alaskan Native (AI/AN) population (estimated 60,000 residents), including five indigenous tribal communities (Goshute, Navajo, Paiute, Shoshone, and Ute).^5^ Notably, the proportion of AI/AN in the state exceeds the corresponding proportion on the national level.^4^ The third largest minority group in Utah is the African American population (about 48,000 residents).^5^ As did Hispanics/Latinos, this population grew rapidly in the most recent census-to-census estimates (by 77%).^5^ In addition, nearly 20% of the Utah African American population was born outside the United States,^5^ some of who are refugees who fled civil wars, genocides, or ethnic cleansing.^6^ Finally, Utah is home to a considerable Native Hawaiian/Pacific Islander (NH/PI) population (nearly 38,000 residents).^5^ The proportion of NH/PI in Utah is much higher than the national average, with one out of four Tongan Americans residing in the state.^5^

Although nationally, Hispanics/Latinos, AI/ANs, African Americans, and Asians have higher rates of HPV vaccine series completion than non-Hispanic whites,^3^ small sample sizes prevent population estimates for these groups in Utah,^7^ and studies of some minority populations in the state have reported severe undervaccination.^8^ Furthermore, the Utah Department of Health has documented disparities in numerous other preventive healthcare procedures (e.g., Pap smears, colonoscopies, and flu shots) among these minority groups.^9^

While prior literature has identified numerous positive correlates of HPV vaccination in the general population—including caregiver age, being female, income, educational attainment, and health insurance coverage^10–15^—it is likely that diverse families in Utah face a unique set of barriers to accessing the HPV vaccine.

To better serve these communities and achieve national HPV vaccination goals,^16^ we need to examine the correlates of HPV vaccination within diverse communities. Therefore, in this study, we worked with five Utah community groups representing African American, African refugee, AI/AN, Hispanic/Latino, and NH/PI populations to probe the facilitators and barriers to HPV vaccination.

## Materials and Methods

This article is part of a larger research project in which adult family members of teens participated in focus groups and surveys about their HPV vaccine-related beliefs and behaviors. This article is focused on the survey results about HPV vaccine receipt and intentions for vaccination. We also explore reported barriers to vaccination.

The larger research project was conducted in collaboration with Community Faces of Utah (CFU), a partnership between five racial/ethnic community organizations (representing African Americans, African refugees, AIs/ANs, Hispanics/Latinos, and NHs/PIs), the Utah Department of Health, and the Collaboration and Engagement Team of the Center for Clinical and Translational Science at the University of Utah. The development, implementation, analysis, and dissemination phases of this study were guided by a community-based participatory research approach; therefore, the CFU community leaders actively participated in developing data collection instruments, recruiting participants, facilitating data collection, interpreting findings, and presenting conclusions at conferences. The study was conducted between May 2014 and February 2015. All research was reviewed and approved by the University of Utah Institutional Review Board.

### Study sample

Participants were adult (aged 18 years or older) parents, legal guardians, or caregivers who were vaccination decision-makers for teens aged 11–17 years. CFU community leaders conducted convenience sampling through their organizational networks to recruit participants in two phases. In the first phase (May 2014–October 2014), participants were recruited to participate in a focus group and complete a survey; they were offered a $25 gift card incentive. In the second phase (October 2014–February 2015), participants were recruited to complete the (same) survey only; they were offered a $15 gift card incentive. This article presents results from the survey data only; other articles present the results from the focus groups.^17^

### Measures

The 21-item survey was developed based on an internal literature review and the input of CFU community leaders. This survey assessed sociodemographic characteristics, acculturation indicators (such as birthplace and English language use), participants' awareness and knowledge of the HPV vaccine, and adolescents' receipt or intended receipt of the HPV vaccine. For receipt and intended receipt of HPV vaccination, participants were asked about adolescent sons and daughters separately, however, the question was not specific to individual daughters and sons if participants had more than one daughter or son. An English version of the survey was used by the African American, AI/AN, and NH/PI community organizations. A Spanish version of the survey was used by the Hispanic/Latino community organization. As the majority of the African refugees did not read in their native language, an English version of the survey was read aloud to participants by community translators and completed using a color-coded schematic. With the exception of the African refugee group, all surveys were self-administered with community translators available to answer questions.

In this article, the primary outcomes of interest are receipt of at least one dose of the HPV vaccine and intentions for HPV vaccination. Additional outcomes of interest are reported barriers to HPV vaccination. These barriers were assessed separately for daughters and sons as well. The sociodemographic correlates assessed (based on an internal literature review and the input of CFU community leaders) were caregiver age, gender, race/ethnicity, marital status, educational attainment, annual household income, occupation, birthplace, caregiver parent birthplace, English usage, health insurance coverage, type of health insurance coverage, and whether adolescents have a primary care provider.

### Statistical analyses

Frequency counts and percentages of selected characteristics are reported for the sample (Table 1). For all demographic variables, Fisher's tests were used to compare distributions of caregivers who had vaccinated any child with at least one dose of the HPV vaccine and those who had not (Table 1). Among caregivers of unvaccinated children, for all demographic variables, Fisher's tests were used to compare distributions between participants who reported being very likely, somewhat likely, or unlikely to vaccinate their daughters or sons (Table 2) with the HPV vaccine. Among caregivers of unvaccinated children, reasons for not vaccinating were assessed (Table 3). Due to issues of complete separation in the categorical predictors, possibly due to small cell sizes, multivariable regression was unable to be completed. For all analyses, *p*-values of <0.05 were considered significant. All statistical analyses were performed using R-2.15.1 (R Development Core Team, 2014).

**Table 1.** **Demographic Characteristics of Participants (*N*=228) and Correlates of Receipt of Human Papillomavirus Vaccine for Any Child Among Caregivers of Children Age 11–17 Years (*N*=181)**

	Total, *n*^a^ (%)^b^	Any child received HPV vaccine (*n*=37), *n* (%)^c^	No child received HPV vaccine (*n*=144), *n* (%)^c^	*p*^d^
Age group, years old				**0.0354**
<35	42 (18.42)	9 (32.14)	19 (67.86)	
35–50	148 (64.91)	27 (21.43)	99 (78.57)	
>50	35 (15.35)	1 (4.17)	23 (95.83)	
Gender				0.1440
Male	64 (28.07)	6 (12.50)	42 (87.50)	
Female	162 (71.05)	31 (23.48)	101 (76.52)	
Race/ethnicity				**0.0003**
African American	17 (7.46)	3 (30.00)	7 (70.00)	
African immigrant	39 (17.12)	1 (3.57)	27 (96.43)	
American Indian/Alaskan Native	23 (10.09)	8 (47.06)	9 (52.94)	
Hispanic/Latino	64 (28.07)	14 (29.79)	33 (70.21)	
Native Hawaiian/Pacific Islander	70 (30.70)	7 (10.61)	59 (89.39)	
Other (includes multiracial)	7 (3.07)	3 (42.86)	4 (57.14)	
Marital status				1.0000
Married or living as married	174 (76.32)	29 (20.57)	112 (79.43)	
Other	49 (21.49)	7 (20.00)	28 (80.00)	
Educational attainment				**0.0055**
<High school	29 (12.72)	0 (0.00)	23 (100.00)	
High school/GED	85 (37.28)	15 (21.74)	54 (78.26)	
AD diploma or certificate	52 (22.81)	10 (25.00)	30 (75.00)	
≥Bachelor's degree	42 (18.42)	12 (36.36)	21 (63.64)	
Annual household income				**0.0454**
<$20,000	86 (37.72)	10 (14.08)	61 (85.92)	
$20,000–$40,000	69 (30.26)	10 (18.87)	43 (81.13)	
>$40,000	60 (26.32)	17 (32.69)	35 (67.31)	
Occupation^e^				0.0698
Service employee	26 (11.40)	4 (18.18)	18 (81.82)	
Business employee	36 (15.79)	11 (35.48)	20 (64.52)	
Building/construction employee	42 (18.42)	2 (6.06)	31 (93.94)	
Other employment	27 (11.84)	6 (30.00)	14 (70.00)	
Student	10 (4.39)	1 (14.29)	6 (85.71)	
Homemaker	40 (17.54)	8 (25.00)	24 (75.00)	
Unemployed/retired	17 (7.46)	1 (8.33)	11 (91.67)	
Birthplace				0.1178
United States	72 (31.58)	16 (27.59)	42 (72.41)	
Other^f,g^	154 (67.54)	21 (17.21)	101 (82.79)	
Years in the United States years^h^				**0.0229**
<20	68 (44.16)	14 (25.45)	41 (74.55)	
≥20	74 (48.05)	5 (8.62)	53 (91.38)	
Parents' birthplace				**0.0084**
United States	47 (20.61)	13 (38.24)	21 (61.76)	
Other	176 (77.19)	24 (16.44)	122 (83.56)	
English usage^i^				0.4077
Low (<2.5)	43 (18.86)	7 (21.88)	25 (78.13)	
Medium (2.5–4)	72 (31.58)	11 (17.74)	51 (82.26)	
High (>4)	61 (26.75)	14 (28.57)	35 (71.43)	
Health insurance coverage				0.7103
Yes	127 (55.70)	23 (21.90)	82 (78.10)	
No	94 (41.23)	14 (19.18)	59 (80.82)	
Type of health insurance^j^				0.1492
Private	82 (64.57)	18 (25.00)	54 (75.00)	
Public	40 (31.50)	5 (12.82)	34 (87.18)	
Primary care provider for child				0.8229
Yes	158 (69.30)	29 (21.80)	104 (78.20)	
No	56 (24.56)	8 (18.60)	35 (81.40)	

Boldface indicates significance (*p*<0.05).

^a^ Missing values are not shown in this table. Missing values are as follows: age group (3 missing, including 1 participant excluded because reported age was “4”; range: 18–74; mean: 43.09; SD: 10.19); gender (2 missing); race/ethnicity (8 missing); marital status (5 missing); educational attainment (20 missing); annual household income (13 missing); occupation (30 missing); birthplace (2 missing); years in the United States (86 missing, including 74 excluded for being born in the United States; range: 2–55; mean: 25.62; SD: 17.25); parents' birthplace (5 missing); English usage (52 missing); health insurance coverage (7 missing); type of health insurance (106 missing, including 94 excluded for not having any health insurance); primary care provider for child (14 missing).

^b^ Percentages calculated out of 228 (number of participants), except where otherwise noted.

^c^ Percentages calculated out of row totals.

^d^ *p*-Values calculated using Fisher's exact test for count data (this test chosen due to small cell sizes).

^e^ Occupation coded using U.S. Standard Occupational Codes and collapsed such that “Service” includes community and social service occupations, protective service occupations, food preparation and serving-related occupations, and personal care and service occupations; “Business” includes management occupations, business and financial operation occupations, architecture and engineering occupations, sales and related occupations, and office and administrative support occupations; “Building/construction” includes building and ground cleaning and maintenance occupations, construction and extraction occupations, installation, maintenance, and repair occupations, production occupations, and transportation and material moving occupations; “Other employment” includes legal occupations, education, training, and library occupations, arts, design, entertainment, sports, and media occupations, healthcare practitioners and technical occupations, and healthcare support occupations.

^f^ Countries of origin: The United States, *n* = 74; Latin American countries, *n* = 65 (Mexico: *n* = 44; Peru: *n* = 6; Guatemala: *n* = 4; Argentina: *n* = 3; El Salvador: *n* = 2; Honduras: *n* = 2; Colombia: *n* = 1; Ecuador: *n* = 1; Venezuela: *n* = 1; Dominican Republic: *n* = 1), Pacific Islands, *n* = 45 (Tonga, *n* = 42; Samoa, *n* = 2; Vanuatu, *n* = 1), African Countries, *n* = 39 (African country not specified, *n* = 2; Burundi *n* = 17; Congo, *n* = 11; Rwanda: *n* = 6; Liberia, *n* = 2; Tanzania, *n* = 1, and Australia, *n* = 3).

^g^ *n* =  60 (93.75%) Hispanic/Latino participants were immigrant and *n* = 4 (6.25%) Hispanic/Latino participants were nonimmigrant; *n* = 47 (67.14%) Native Hawaiian/Pacific Islander participants were immigrant and *n* = 23 (32.86%) Native Hawaiian/Pacific Islander participants were nonimmigrant; *n* = 1 (14.29%) other/multiracial participants were immigrant, and *n* = 6 (85.71%) other/multiracial participants were nonimmigrant. HPV, human papillomavirus; SD, standard deviation.

^h^ Percentages calculated out of 154 (No. of participants reported born out of the United States).

^i^ Five questions were used to create this composite score. Participants answered these questions according to a 5-point scale, and responses were coded numerically (1–5). To create the composite score, numeric values associated with the participants' responses to each of the five questions were added and then divided by the number of questions that the participant answered. Essentially, each participant's responses to the five English usage-related questions were averaged.

^j^ Percentages calculated out of 127 (number of participants reported having health insurance).

HPV, human papillomavirus; SD, standard deviation.

**Table 2.** **Demographic Correlates of Caregivers' Intention to Vaccinate Unvaccinated Sons (*N*=100) and Daughters Age 11–17 Years with the Human Papillomavirus Vaccine (*N*=99)**

	Sons				Daughters			
	Very likely (*n*=20), *n* (%)^a^	Somewhat likely (*n*=11), *n* (%)^a^	Unlikely (*n*=11), *n* (%)^a^	*p*^b^	Very likely (*n*=14), *n* (%)^a^	Somewhat likely (*n*=5), *n* (%)^a^	Unlikely (*n*=13), *n* (%)^a^	*p*^b^
Age group, years old				0.8902				1.0000
<35	1 (25.00)	1 (25.00)	2 (50.00)		3 (42.86)	1 (14.29)	3 (42.86)	
35–50	13 (50.00)	7 (26.92)	6 (23.08)		10 (45.45)	3 (13.64)	9 (40.91)	
>50	5 (45.45)	3 (27.27)	3 (27.27)		0 (0.00)	0 (0.00)	1 (100.00)	
Gender				0.0642				0.0322
Male	7 (70.00)	3 (30.00)	0 (0.00)		5 (83.33)	1 (16.67)	0 (0.00)	
Female	12 (38.71)	8 (25.81)	11 (35.48)		8 (32.00)	4 (16.00)	13 (52.00)	
Race/ethnicity				**0.0025**				**0.0480**
African American	1 (25.00)	3 (75.00)	0 (0.00)		0 (0.00)	0 (0.00)	0 (0.00)	
African immigrant	0 (0.00)	0 (0.00)	0 (0.00)		1 (100.00)	0 (0.00)	0 (0.00)	
American Indian/Alaskan Native	1 (25.00)	2 (50.00)	1 (25.00)		1 (50.00)	0 (0.00)	1 (50.00)	
Hispanic/Latino	12 (80.00)	2 (13.33)	1 (6.67)		8 (61.54)	2 (15.38)	3 (23.08)	
Pacific Islander	5 (29.41)	3 (17.65)	9 (52.94)		2 (15.38)	2 (15.38)	9 (69.23)	
Other (includes multiracial)	0 (0.00)	1 (100.00)	0 (0.00)		0 (0.00)	1 (100.00)	0 (0.00)	
Marital status				0.8378				0.4633
Married or living as married	18 (48.65)	10 (27.03)	9 (24.32)		12 (48.00)	3 (12.00)	10 (40.00)	
Other	2 (40.00)	1 (20.00)	2 (40.00)		2 (28.57)	2 (28.57)	3 (42.86)	
Educational attainment				0.4339				0.1537
<High school	2 (66.67)	1 (33.33)	0 (0.00)		3 (100.00)	0 (0.00)	0 (0.00)	
High school/GED	6 (42.86)	3 (21.43)	5 (35.71)		3 (27.27)	2 (18.18)	6 (54.55)	
AD diploma or certificate	8 (66.67)	2 (16.67)	2 (16.67)		7 (63.64)	1 (9.09)	3 (27.27)	
≥Bachelor's degree	3 (25.00)	5 (41.67)	4 (33.33)		1 (14.29)	2 (28.57)	4 (57.14)	
Annual household income				0.5340				0.6834
<$20,000	6 (54.55)	1 (9.09)	4 (36.36)		5 (38.46)	2 (15.38)	6 (46.15)	
$20,000–$40,000	9 (52.94)	5 (29.41)	3 (17.65)		7 (58.33)	2 (16.67)	3 (25.00)	
>$40,000	5 (35.71)	5 (35.71)	4 (28.57)		2 (28.57)	1 (14.29)	4 (57.14)	
Occupation^c^				0.4062				0.6556
Service employee	3 (75.00)	1 (25.00)	0 (0.00)		2 (66.67)	0 (0.00)	1 (33.33)	
Business employee	7 (53.85)	3 (23.08)	3 (23.08)		3 (60.00)	1 (20.00)	1 (20.00)	
Building/construction employee	3 (75.00)	1 (25.00)	0 (0.00)		3 (100.00)	0 (0.00)	0 (0.00)	
Other employment	2 (33.33)	2 (33.33)	2 (33.33)		2 (50.00)	0 (0.00)	2 (50.00)	
Student	0 (0.00)	0 (0.00)	0 (0.00)		0 (0.00)	0 (0.00)	0 (0.00)	
Homemaker	2 (40.00)	2 (40.00)	1 (20.00)		3 (37.50)	2 (25.00)	3 (37.50)	
Unemployed/retired	1 (20.00)	0 (0.00)	4 (80.00)		0 (0.00)	0 (0.00)	2 (100.00)	
Birthplace				0.2371				0.4247
United States	5 (31.25)	6 (37.50)	5 (31.25)		2 (25.00)	2 (25.00)	4 (50.00)	
Other	15 (57.69)	5 (19.23)	6 (23.08)		12 (50.00)	3 (12.50)	9 (37.50)	
Years in the United States				0.0561				1.0000
<20	8 (66.67)	0 (0.00)	4 (33.33)		4 (40.00)	2 (20.00)	4 (40.00)	
≥20	5 (41.67)	5 (41.67)	2 (16.67)		5 (50.00)	1 (10.00)	4 (40.00)	
Parents' birthplace				**0.0232**				1.0000
United States	2 (18.18)	6 (54.55)	3 (27.27)		1 (100.00)	0 (0.00)	0 (0.00)	
Other	18 (58.06)	5 (16.13)	8 (25.81)		13 (43.33)	5 (16.67)	12 (40.00)	
English usage^c^				0.7659				0.4599
Low (<2.5)	4 (57.14)	1 (14.29)	2 (28.57)		4 (66.67)	0 (0.00)	2 (33.33)	
Medium (2.5–4)	5 (45.45)	4 (36.36)	2 (18.18)		4 (26.67)	4 (26.67)	7 (46.67)	
High (>4)	5 (33.33)	5 (33.33)	5 (33.33)		2 (50.00)	1 (25.00)	1 (25.00)	
Health insurance coverage				**0.0465**				**0.0281**
Yes	7 (33.33)	9 (42.86)	5 (23.81)		4 (22.22)	4 (22.22)	10 (55.56)	
No	13 (61.90)	2 (9.52)	6 (28.57)		10 (71.43)	1 (7.14)	3 (21.43)	
Type of health insurance				**0.0079**				0.3277
Private	7 (36.84)	9 (47.37)	3 (15.79)		2 (15.38)	4 (30.77)	7 (53.85)	
Public	0 (0.00)	0 (0.00)	4 (100.00)		2 (40.00)	0 (0.00)	3 (60.00)	
Primary care provider for child				0.6932				0.2293
Yes	15 (45.45)	10 (30.30)	8 (24.24)		8 (33.33)	4 (16.67)	12 (50.00)	
No	4 (50.00)	1 (12.50)	3 (37.50)		4 (66.67)	1 (16.67)	1 (16.67)	

Missing values for variables are not shown in this table and were excluded from analysis. See Table 1 for counts of missing values for each predictor variable.

Boldface indicates significance (*p*<0.05).

^a^ Percentages calculated out of row totals.

^b^ *p*-Values calculated using Fisher's exact test for count data (this test was chosen due to small cell sizes).

^c^ See Table 1 for explanation of variable creation.

**Table 3.** **Summary Statistics for Reasons for Not Vaccinating Children Among Caregivers of Unvaccinated Children Age 11–17 Years (*N*=144)**

	Yes, *n* (%)^a^	No, *n* (%)^a^
Daughters (*n*=99)		
Didn't know about the HPV vaccine	52 (52.53)	21 (21.21)
Not the right age	10 (10.10)	63 (63.64)
Not recommended	4 (4.04)	69 (69.70)
It is unnecessary	12 (12.12)	61 (61.62)
She is not sexually active	13 (13.13)	60 (60.61)
It will promote sexual activity	3 (3.03)	70 (70.71)
Side effects	14 (14.14)	59 (59.60)
Costs	18 (18.18)	55 (55.56)
I don't vaccinate my children	7 (7.07)	66 (66.67)
Other	5 (5.05)	68 (68.69)
Sons (*n*=100)		
Didn't know about the HPV vaccine	52 (52.00)	30 (30.00)
Not the right age	11 (11.00)	71 (71.00)
Not recommended	6 (6.00)	76 (76.00)
It is unnecessary	12 (12.00)	70 (70.00)
He is not sexually active	22 (22.00)	60 (60.00)
It will promote sexual activity	0 (0.00)	82 (82.00)
Side effects	18 (18.00)	64 (64.00)
Costs	18 (18.00)	64 (64.00)
I don't vaccinate my children	3 (3.00)	79 (79.00)
Other	11 (11.00)	71 (71.00)

Missing values for variables are not shown in this table and were excluded from analysis. It is possible to calculate missing values for each reason using simple subtraction.

^a^ Percentages calculated out of total number of daughters or sons (i.e., 99 for daughters, 100 for sons).

## Results

There were 228 participants; 93 were recruited during the first phase of data collection (completing a focus group and a survey), and 135 returned surveys during the second phase of data collection.

### Demographic characteristics

Demographic characteristics of participants are presented in Table 1. The majority of participants were 35–50 years old (*n*=148, 64.91%), female (*n*=162, 71.05%), married or living as married (*n*=174, 76.32%), and born out of the United States (*n*=154, 67.54%). Free text responses of country of origin for those who provided responses showed that *n*=74 (32.74%) reported being born in the United States, *n*=65 (28.76%) reported being born in Latin American countries, *n*=45 (19.91%) being born in Pacific Islands, *n*=39 (17.26%) from African countries, and *n*=3 (1.33%) from Australia (a list of participants' countries of origin is provided in the footnotes of Table 1). Among participants born out of the United States, there was a nearly even split between those who had spent fewer than 20 years and those who had spent more than 20 years in the United States (*n*=68, 44.16% vs. *n*=74, 48.05%). Most participants' parents were also born out of the United States (*n*=176, 77.19%). Despite the large proportion of participants who were immigrants, there was a fairly even distribution across English use (18.86% low, 31.58% medium, and 26.75% high). Most participants had health insurance (*n*=127, 55.70%) and had a primary care provider for their children (*n*=158, 69.30%). Among participants who reported having health insurance, the majority had private insurance (*n*=82, 64.57%).

### HPV vaccination

About 20% of participants had self-reported at least one of their children receiving a minimum of one dose of the HPV vaccine (Table 1). In bivariate analyses (Table 1), caregiver age (*p*=0.0354), race/ethnicity (*p*=0.0003), educational attainment (*p*=0.0055), annual household income (*p*=0.0454), years in the United States (*p*=0.0229), and caregiver parent birthplace (*p*=0.0084) were all significantly associated with HPV vaccination.

### Intentions for HPV vaccination

In bivariate analyses of participants with unvaccinated daughters (*N*=144, Table 2), race/ethnicity (*p*=0.0480) and health insurance coverage (*p*=0.0281) were significantly associated with intention to vaccinate daughters. In bivariate analyses of participants with unvaccinated sons (Table 2), race/ethnicity (*p*=0.0025), caregiver parent birthplace (*p*=0.0232), health insurance coverage (*p*=0.0465), and type of health insurance coverage (*p*=0.0079) were significantly associated with intention to vaccinate sons.

### Barriers to HPV vaccination

Participants were asked to select the top three barriers to HPV vaccination for the daughters and sons. Among caregivers of unvaccinated children (*N*=144), reasons for not vaccinating were assessed (Table 3). The top 3 reasons given for not vaccinating eligible daughters were as follows: not knowing about the HPV vaccine (*n*=52, 52.53%), costs (*n*=18, 18.18%), and side effects (*n*=14, 14.14%). The top 4 reasons given for not vaccinating eligible sons were as follows: not knowing about the HPV vaccine (*n*=52, 52.00%), son not being sexually active (*n*=22, 22.00%), costs (*n*=18, 18.00%), and side effects (*n*=18, 18.00%). Notably, very few caregivers reported concerns that the HPV vaccine would promote sexual activity (*n*=3 for daughters, *n*=0 for sons; see Table 3 for counts and percentages for all barriers).

## Discussion

The HPV vaccine can prevent a variety of HPV-related diagnoses and deaths^2^; yet coverage remains low in the United States, and even lower in Utah.^3^ Given the documented disparities in other preventive healthcare among minority groups in Utah,^9^ it is possible that diverse communities in the state also fare comparatively worse in HPV vaccination. In this study, we examined the sociodemographic correlates of HPV vaccination among five diverse populations in Utah: African Americans, African refugees, American Indians/Alaskan Natives, Hispanics/Latinos, and Native Hawaiians/Pacific Islanders. To the best of our knowledge, this is the first study of HPV vaccination outcomes among these five populations in a state with a unique racial/ethnic history.

### HPV vaccination

Only about 20% of participants had vaccinated at least one of their children with at least one dose of the HPV vaccine—a dramatically lower figure than national estimates (60%), including national estimates associated with specific racial/ethnic groups (black teens, 66%; Hispanic teens, 70%; AI/AN, 62%; Asian teens, 63%; multiracial teens, 61%).^3^ This finding signals the need for immediate interventions to serve these diverse communities in Utah.

We found that caregiver age was significantly related to HPV vaccination, with younger participants composing a higher percentage of those who vaccinated their children than those who did not. This finding supports the previous literature that has consistently shown younger parent age (younger than 35 years) to be associated with children's HPV vaccine receipt^10,18^ in the region and nationally, and suggests that interventions should target older parents (older than 35 years) in diverse communities, perhaps through platforms more frequently used by this demographic (e.g., radio).^17,19^

Our results also showed that race/ethnicity, number of years in the United States, and caregiver parent birthplace were significantly related to HPV vaccination. The finding of differences in HPV vaccination by racial/ethnic group illustrates that there is no one-size-fits-all solution for these populations; rather, each minority group will require a unique set of tailored intervention strategies. While a larger percentage of participants who vaccinated their children had U.S.-native parents, among immigrant participants, a larger percentage of those who lived in the United States for fewer than 20 years had vaccinated their children compared with those who lived in the United States for more than 20 years. These findings highlight the “immigrant paradox,”^20^ and suggest that in addition to tailoring interventions to each racial/ethnic group, it will be necessary to give particular attention to immigrants, regardless of how long they have lived in the United States.

Finally, our findings included significant negative associations between HPV vaccination and both educational attainment and annual household income. We found that a larger percentage of participants who did not vaccinate their children had lower educational attainment and lower annual household income. These findings support previous literature documenting the same associations across many populations,^10,12,13,15^ and imply that HPV vaccination interventions should devote focus to these at-risk families.

### Intentions for HPV vaccination

We found that among parents with unvaccinated children, race/ethnicity and caregiver parent birthplace were significantly related to intentions to vaccinate. We found that a greater proportion of Hispanic/Latino caregivers were willing to vaccinate their unvaccinated sons and daughters with the HPV vaccine compared with the other racial/ethnic groups. Comparatively, willingness to vaccinate sons and daughters was lower in the NH/PI community. A recent study comparing Hispanic/Latino and Asian and Pacific Islander parents found that while the awareness of the HPV vaccine increased most among Hispanic/Latino mothers between 2007 and 2011, Asian or Pacific Islander mothers continued to have lower awareness.^21^

In addition, a greater proportion of caregivers' whose parents were born outside of the United States indicated greater willingness to vaccinate their unvaccinated sons in the next 12 months. However, willingness to vaccinate was not significant for daughters. As with HPV vaccination, these findings support the previous literature documenting racial/ethnic and immigrant/native differences^11^ and signal the need for interventions to be tailored to each racial/ethnic community group and to be sensitive to immigrants and first-generation Americans.

We also found that having health insurance coverage was significantly related to intentions to vaccinate sons and daughters with the HPV vaccine, which is supported by other studies as well.^22,23^ In addition, we found that having private insurance was associated with willingness to vaccinate sons but not daughters. Although the mechanisms underlying these findings are not fully understood, campaigns to raise awareness of low-cost or free HPV vaccination through local healthcare providers (e.g., through the Vaccines for Children program) may payoff.

### Barriers to HPV vaccination

The most frequently reported reason for not vaccinating male and female children was lack of knowledge about the HPV vaccine, which indicates the continued need for raising awareness of the existence of the vaccine, which is supported by other research.^21^ Racial and ethnic minorities are at greater risk for lower awareness about the HPV vaccine, suggesting the need for educational interventions among diverse communities.^24^ These efforts could take the form of educational media campaigns^17,25,26^ or collaborations with clinicians.^27^ The next most frequently reported barrier to vaccinating both sons and daughters was the cost of the vaccine, despite the availability of low-cost or free HPV vaccinations through the Vaccines for Children program. This finding suggests that caregivers may not be receiving the necessary information about these programs, so efforts at the community level, such as patient navigation programs or health fairs, may help.^28,29^

Another top barrier common to both sons and daughters was the concern about side effects. Unlike the barriers of not knowing about the HPV vaccine and of perceived costs of the HPV vaccine, these barriers suggest that parents may be aware of the existence and availability of the HPV vaccine, but are misinformed about its safety and recommendation guidelines. Therefore, providers and community organizations should focus education efforts on the safety profile of the HPV vaccine and emphasize the benefits of early vaccination. Finally, a top barrier more common to caregivers of unvaccinated sons, which was the second-most frequently reported barrier for this group, was the perception that sons were not yet sexually active. This finding suggests that diverse caregivers of sons may have greater informational needs about the HPV vaccine recommendations, which has been described as a risk factor for lower awareness of the HPV vaccine in other studies.^21^

### Limitations

This study is limited by the use of self-reported HPV vaccination; it is possible that actual HPV vaccination rates for these populations are higher or lower than those we report; a study by Dorell et al. on parental recall of vaccination status for children in the National Immunization Survey Teen dataset found that underreporting HPV vaccination status (16.6%) was more common than overreporting (7.8%) when comparing parental recall versus medical record data.^30^ Moreover, we did not distinguish between HPV vaccine initiation (at least one dose) and completion (all three doses). Another limitation of the study is the use of purposive sampling. Participants selected for the study may have been more active in their racial/ethnic community groups than other members of the populations. In addition, analyses were limited to a bivariate approach, so results do not control for confounding variables and do not assess interaction effects (e.g., between race/ethnicity and other sociodemographic characteristics). Finally, our findings may not be generalizable to other geographic areas with different racial/ethnic history.

## Conclusions

To the best of our knowledge, this is the first study to assess the sociodemographic correlates of HPV vaccination of minority children in Utah, representing African American, African refugee, AI/AN, Hispanic/Latino, and NH/PI communities. Our findings support the need for community approaches to HPV education among diverse caregivers in Utah, particularly the need to better inform communities about the availability of low-cost or free HPV vaccines and about the benefits of the HPV vaccine, and to clarify misunderstandings about the vaccine. Our results also indicate target audiences—groups that are at particular risk for not vaccinating their adolescents—of older, immigrant, and socioeconomically disadvantaged caregivers. Furthermore, we recommend further research to develop targeted interventions for the NH/PI communities and diverse caregivers of vaccine-eligible sons.

Finally, we recognize that the most vulnerable community of this sample, the African refugee community, may be affected by specific factors influencing HPV vaccination that are not directly assessed within the scope of this study; therefore, we recommend that future research relevant to this population be conducted.

## Compliance with Ethical Standards

### Ethical approval

All procedures performed in studies involving human participants were in accordance with the ethical standards of the institutional and/or national research committee and with the 1964 Declaration of Helsinki and its later amendments or comparable ethical standards.

### Informed consent

Informed consent was obtained from all individual participants included in the study.

## Abbreviations Used

AI/AN: American Indian/Alaskan Native
CFU: Community Faces of Utah
HPV: human papillomavirus
NH/PI: Native Hawaiian/Pacific Islander
